# HTLV-3 and HTLV-4 antisense proteins enhance the transactivation potential of several Jun family members through interaction via their bZIP-like domain

**DOI:** 10.1186/1742-4690-11-S1-O72

**Published:** 2014-01-07

**Authors:** Émilie Larocque, Charlotte André-Arpin, Jean-Michel Mesnard, William M Switzer, Benoit Barbeau

**Affiliations:** 1 Département des Sciences Biologiques and Centre de Recherche BioMed, Université du Québec à Montréal, Montréal (Québec) Canada; 2 Université Montpellier 1, Centre d’Etudes d’Agents Pathogènes et Biotechnologies pour la Santé, CNRS, UM5236, Montpellier, France; 3 Laboratory Branch, Division of HIV/AIDS Prevention, National Center for HIV, STD, and TB prevention, Centers for Disease Control and Prevention, Atlanta, GA, USA

## 

HTLV-3 and HTLV-4 are two recently identified viruses closely related to HTLV-1, which have not yet been linked to any diseases. Similarly to HTLV-1 and HTLV-2, which both express antisense transcript-derived HBZ and APH-2, respectively, HTLV-3 and HTLV-4 harbor antisense genes termed APH-3 and APH-4. These two encoded proteins contain an atypical bZIP domain but are still able to down-regulate Tax-mediated LTR activation, alike HBZ and APH-2. Since HBZ and APH-2 affect Jun-dependent transcription differently, our goal was to assess the impact of APH-3 and APH-4 on the transactivation potential of Jun family members. Co-IP experiments first showed that both APH-3 and APH-4 interacted with all tested Jun members. Jun/APH complexes significantly enhanced Jun-dependent transactivation of the human collagenase promoter containing a single AP-1 binding site as well as a construct bearing a minimal promoter and AP-1-binding sites. APH-3 and APH-4 deletion mutants and point mutations of specific leucine residues demonstrated that this functional interaction was mediated by the bZIP-like domain. Using a construct with a minimal promoter bearing GAL4-binding sites and APH-3/APH-4 expression vectors fused to the GAL4 DNA binding domain, we further showed that, in contrast to HBZ, APH-3 and APH-4 did not contain an activation domain. These results highlight the varying capacity of different HTLV-encoded antisense proteins to act upon transactivation mediated by Jun transcription factors. Taking into account the previously reported association between HBZ and ATL development, determining the functional differences of these HTLV antisense proteins could contribute in better understanding this association.

